# Challenges of the management of mass casualty: lessons learned from the Jos crisis of 2001

**DOI:** 10.1186/1749-7922-8-44

**Published:** 2013-10-28

**Authors:** Kenneth N Ozoilo, Ishaya C Pam, Simon J Yiltok, Alice V Ramyil, Hyacinth C Nwadiaro

**Affiliations:** 1 Surgery Department, Jos University Teaching Hospital, Jos, Nigeria; 2 Obstetrics and Gynaecology Department, Jos University Teaching Hospital, Jos, Nigeria; 3 Ophthalmology Department, Jos University Teaching Hospital, Jos, Nigeria

**Keywords:** Challenges, Crisis, Disaster, Mass casualty, Trauma

## Abstract

**Background:**

Jos has witnessed a series of civil crises which have generated mass casualties that the Jos University Teaching Hospital has had to respond to from time to time. We review the challenges that we encountered in the management of the victims of the 2001 crisis.

**Methodology:**

We reviewed the findings of our debriefing sessions following the sectarian crisis of September 2001 and identified the challenges and obstacles experienced during these periods.

**Results:**

Communication was a major challenge, both within and outside the hospital. In the field, there was poor field triage and no prehospital care. Transportation and evacuation was hazardous, for both injured patients and medical personnel. This was worsened by the imposition of a curfew on the city and its environs. In the hospital, supplies such as fluids, emergency drugs, sterile dressings and instruments, splints, and other consumables, blood and food were soon exhausted. Record keeping was erratic. Staff began to show signs of physical and mental exhaustion as well as features of anxiety and stress. Tensions rose between different religious groups in the hospital and an attempt was made by rioters to attack the hospital. Patients suffered poor subsequent care following resuscitation and/or surgery and there was neglect of patients on admission prior to the crisis as well as non trauma medical emergencies.

**Conclusion:**

Mass casualties from disasters that disrupt organized societal mechanisms for days can pose significant challenges to the best of institutional disaster response plans. In the situation that we experienced, our disaster plan was impractical initially because it failed to factor in such a prolongation of both crisis and response. We recommend that institutional disaster response plans should incorporate provisions for the challenges we have enumerated and factor in peculiarities that would emanate from the need for a prolonged response.

## Introduction

In a mass casualty situation, there is a sudden presentation of large numbers of injured people at a rate that exceeds the capacity of the institution to cope [1]. Traditional institutional response to such situations involves expanding of the surge capacity by mobilizing additional resources from within the hospital to provide care for the injured patients [2]. This involves mobilization of staff from other parts of the hospital to the accident and emergency department and a call out system for staff that are outside the hospital [3]. A slight diminution in standard of care will also be endured in which trauma care assets are diverted from less critically injured patients to more critically injured, but salvageable patients [4]. Sometimes help might be sought from other hospitals within and outside the region [2]. This works well when there is a one-off event, and preservation of organized societal mechanisms permitting flow of supplies, personnel and other aid to and from the hospital. When there is ongoing hostility, involving the whole city, and lasting several days, new challenges emerge which interfere with this mobilization of resources from within and outside the hospital. This undermines efforts at mounting an effective response to the disaster situation. On the 7th of September 2001, Jos, the capital Plateau state of Nigeria witnessed a sectarian crisis which lasted for five days and generated several injured patients which presented to our hospital the Jos University Teaching Hospital as mass casualties. We present challenges faced in the management of this mass casualties.

## Methodology

Following the resolution of the crisis we held debriefing sessions to assess our overall response to the crisis and identify challenges that were encountered. Participants at each session included all heads of departments and units involved in the response. All doctors and nurses who were part of the effort were also present as were key staff especially those who had been trapped in the hospital for days at a stretch.

We examined patient records from case notes, Accident and Emergency unit records, operating theatre records and our crisis registry. We also gathered information from the firsthand account of those who were actively involved in the response.

The challenges encountered were catalogued and possible solutions were suggested. The summary of the sessions was compiled and referred to the hospital disaster committee for incorporation into the hospital disaster plan.

## Results

Available records showed that 463 patients were registered in the hospital over four days giving an average of about 115 patients per day. They presented in surges however and the highest surges were on days 2 and 3 with fewer patients seen on days 1 and 4. Some patients were attended to without being registered. Of those that were registered, the records of 74 were not available, leaving that of only 389 for analysis. There were 348 (89.5%) males and the median age was 26 years.

Table 1 shows the mechanisms of injury with the most common being gunshot in 203 patients (52.2%) and cuts from machetes and knives in 161 patients (41.4%). Table 2 shows the distribution of the injuries by body part, the most frequently affected being the head and neck in 171 patients (44.0%) and the extremities 168 patients (43.2%). Some patients had injury by multiple mechanisms and sustained injuries to multiple body parts.

**Table 1:** Mechanisms of injury

**Mechanism**		**No**	**%**
Penetrating			
	Gunshot	203	52.2
	Machete/knife cuts	161	41.4
	Arrow impalements	14	3.6
Blunt			
	Clubs/sticks	44	11.3
Burns			
	Flame	7	1.8
Total		429	100*

*: Some patients had injury by multiple mechanisms.

**Table 2:** Body parts injured

**Body part**	**No**	**%**
Head/neck	171	44.0
Extremity	168	43.2
Abdomen/pelvis	65	16.7
Chest	30	7.7
Total	434	100*

*: Some patients had injury to multiple body parts.

Table 3 summarizes the challenges encountered in the response to the crisis. Communication was a major challenge, both within and outside the hospital and for collaboration with other agencies responding to the crisis. Field challenges included the violence on the streets, the lack of field triage and the absence of pre-hospital care. Within the hospital, supplies of consumables were quickly exhausted, record keeping was poor, and exhausted staff began to show signs of strain. Hospital safety became threatened at a point both from rising tensions within the premises and from threat of attack from outside. Some patients suffered suboptimal care for reasons ranging from exhaustion of hospital supplies to being forgotten in the heat of the crisis response.

**Table 3:** Challenges encountered

**Communication**		
	Internal	
	External	
	With other agencies	
Field challenges		
	No triage	
	No pre-hospital care	
	Hazard to medical personnel	
Hospital challenges		
	Exhaustion of supplies	
		Intravenous fluids
		Drugs
		Sterile dressings
		Sterile instruments
		Blood
	Poor record keeping	
		Non registration
		Non documentation
		Incomplete documentation
	Staff exhaustion	
		From fatigue/overwork
		Anxiety/tension
	Hospital safety	
		Rising tensions within
		Threat of attack from outside
	Suboptimal patient care	
		From exhaustion of supplies
		Forgotten patients
		Non trauma patients
		Patients on admission prior to onset of crisis

## Discussion

The lack of communication between our hospital and the field meant that we were totally caught unawares at the onset of the crisis. Our first inkling was in the arrival of the first surge of wounded patients. Normal hospital response to severe trauma begins with trauma team activation following advance notification. This is the ideal in isolated trauma scenarios but is even more imperative in mass casualty scenarios. Communication has been identified as a key component of disaster preparedness and response. An analysis of the response to three sequential aircraft crashes in Texas, found communication to be one of the major problems encountered in the implementation of the community and hospital disaster plan [5]. Its total absence meant that we were completely unprepared to receive the first surge of casualties and each subsequent surge was without advance warning. Communication was also needed for mobilizing personnel and other resources from within and outside the hospital, and for information and media management as well as the coordination of response efforts between medical personnel and other agencies of government involved in the disaster response such as the police, military, Red Cross, and other voluntary organizations. The lack of this communication made the overall response efforts disjointed and uncoordinated. The crisis took place before the introduction of mobile telephony in our city and we do not have pagers or two way radios. The existing hospital intercom system and the fixed lines proved grossly inadequate for the internal and external communication needs respectively.

Field triage was crude and did not follow any organized systems. Injured patients were merely conveyed to the hospital if they were fortunate enough to chance upon a military patrol, aid workers and volunteers, or other good Samaritans who were willing and able to help. The aim of triage is to identify that minority of critically injured patients, out of the large pool of patients with less severe injuries so that trauma care assets can be prioritized in favor of the former. Effective triage is necessary to screen out the majority of non critically injured survivors, and results are best when performed by a trained physician in the field [6]. A change in philosophy occurs in the approach to the management of mass casualty: the goal is to do the ‘greatest good for the greatest number’ and not the greatest good for the individual [2,7]. Most effective triage systems accept an overtriage rate of up to 50%, i.e. patients who have been triaged as having critical injuries when in fact they had less severe injuries. This high rate is necessary to reduce the undertriage rate to below 0.5%, i.e. the proportion of patients who were triaged as having non critical injuries when in fact they had critical injuries [7]. In the absence of systematic field triage, a high proportion of patients brought to our facility had non critical injuries as every injured patient was evacuated to the hospital. Higher overtriage rates paradoxically, increase the critical mortality by putting an avoidable strain on the resources needed to manage the critically injured and is therefore undesirable [8].

The absence of a trauma system in our setting meant that there was no prehospital care. It is therefore reasonable to expect that preventable deaths must have occurred in the field. Chances of survival following injuries depend on how fast the patient can be evacuated to a facility that is able to provide treatment for their injuries.

Movement in the field was hazardous for victims, medical personnel and even the military. For this reason, it was extremely difficult to mobilize staff to the hospital to relieve those that were over-worked; in any case, it was not possible for staff that had been at work for several hours at a stretch to go home for the same reason. Some personnel were on ground for 72 to 96 hours without relief. Evacuation of the casualties was left mainly to security personnel. Non military personnel who carried out rescue did so at great personal risk. Some medical personnel who braved the streets were attacked, and when a 24 hour curfew was imposed on the city and its environs, such attacks were as likely to come from military personnel enforcing the curfew as they were to come from rioting civilians breaking it.

There was a lag in the take off of the hospital response, due to lack of prior warning. Once it started however, it was efficient in the first 24 to 48 hours. Subsequently supplies began to run out with a resultant dip in the standard of care. Intravenous fluids, dressing material, splints, essential drugs, sterile instruments and blood soon ran out. We noted particularly that patients requiring large volumes of blood transfusion for resuscitation in the ER often depleted the blood bank reserves without surviving, in the process putting a huge strain on the availability of the product for those that required it for surgical operations. This explains why some protocols urge that serious consideration be given to avoiding blood transfusion in such situations [9].

Supplies had been mobilized from other parts of the hospital as the ER reserves ran low, but it was not possible to replenish these sources as they became exhausted. Even when certain supplies were available in the main hospital store, the myriad of challenges made their availability impossible. For example, while the ER and wards had run out of supplies of sterile dressing materials, the main hospital store had enough stock to last 90 days. These were not available however because the head of stores who had access and authority to release them was not on the premises. Communicating with him was a challenge. When contact was established, he could not come because of the violence in his neighborhood. There was a pool of duty vehicles to convey him, but most drivers were not on the premises and couldn’t come in either. When a driver was mobilized, he required security personnel for protection. The mandate, and preoccupation, of the security personnel of course, was maintenance of law and order, not escort. Such was the nature of the largely logistic problems encountered. The food supplies of the hospital were soon depleted too because not only patients had to be fed, but all people taking refuge in the hospital.

Record keeping was haphazard. Some patients had no medical records. Some had but these were incomplete. Personnel who attended to patients with trivial injuries often moved on to other patients without documenting. Only those who went on to have surgery had detailed and accurate documentation of their treatment. Poor record keeping is ubiquitous in the management of mass casualties but accurate record keeping ensures continuity of care, avoids duplication of efforts, and allows a retrospective analysis of the response effort at debriefing [2,7]. It is recommended that tags (which may be laminated) should be used for identification and teams trained to use short forms and concise writing in keeping patient records under such situations [1,7].

Hospital personnel who were trapped in the hospital for over 72 hours soon began to manifest features of physical and mental stress. Overwork was a major factor, but in addition, there was anxiety for personal safety, fear for the lives of loved ones, and worry over the eventual outcome of the crisis. The sight of severely injured casualties often with grotesque wounds, and the charred, dismembered corpses deposited on the floor outside the morgue (the morgue itself was filled beyond capacity) contributed to the stress. Some people too had narrowly escaped death at the hands of rampaging mobs, prior to finding refuge in the hospital. Acute stress disorders and have been known to accompany the experiencing of such traumatic events and could be a forerunner of Post Traumatic Stress Disorder (PTSD). Although more commonly described among survivors (direct victims) of disasters [2], it has been found among indirect victims such as first responders and the general public [10] and the need for disaster plans to incorporate provisions for emotional evaluation and rehabilitation of casualties is increasingly advocated [2,7].

The Jos crisis of 2001 was in part a religious one. Tensions flared periodically between Christians and Muslims on the premises, due to the mixed composition of the large numbers of people seeking refuge there. Most people, including personnel invariably found their sentiments swayed to on one side of the divide or the other and the ensuing tension threatened to degenerate into violence. It took the dexterity of top management and senior staff to douse the tensions and focus all efforts on the emergency response while emphasizing the need to maintain neutrality in the hospital. Despite this, rumors that victims identified with a particular section were being discriminated against led to an attempt by some rioters to attack the hospital. The perimeter fence of the hospital was already breached before attack was repelled by military personnel guarding the premises. Work place violence is a well documented phenomenon even in peacetime [11-13]. Whether caused by the strain of the ER environment on the staff, or unmet patient expectations, aggression is ultimately fuelled by perception, intolerance, misunderstanding and loss of control [12]. Some patient expectations maybe unrealistic in the ER environment and some of it may be caused by the media. In our case some of the perceptions about the crisis were due to rumours, inaccurate information and faulty reportage by the media. Eruption of violence in the hospital would have brought all response efforts to a halt. Such a situation where the hospital is unable to render any meaningful care to casualties, either because it is itself, consumed by the event (such as war, earthquake or nuclear disaster) or because it is overwhelmed by the sheer volume of casualties, has been termed a Major Medical Disaster [2] and is a situation best prevented.

In the heat of the response, patients who had been transferred to the wards following resuscitation in the ER or operation in the OR often had suboptimal subsequent care. This was because attention was focused on the fresh casualties from the continuing influx in the ER at the expense of those said to have been already “stabilized”. The trickle of personnel who were mobilized from outside the hospital as the crises progressed were directed to the ER and OR, leading to neglect of those in the wards. Some of such patients missed their antibiotics, fluids and wound reviews. Some carried nasogastric tubes and catheters for too long and went for unnecessarily long periods on *nil per os*. There was near total neglect of patients who were on admission in the wards for other reasons prior to the onset of the crisis. Initial response involved mobilization of personnel from the wards to the ER and this did not begin to reverse till near the end of the crisis, five days later.

A unique, if rare category of patients who suffered suboptimal care during this crisis were patients who, developing a medical emergency at home, were able to get to the hospital. Examples include patients with diabetic crises, hypertensive emergencies and other medical emergencies. The care of the trauma patients was prioritized above these patients even when the injuries were not nearly as life threatening. A major contributory factor was the near total absence of internists as part of the disaster response in the erroneous belief that a mass casualty situation called for the mobilization of only surgeons. Some protocols propose that hospital call-in plans should focus on doctors in the surgical specialties and that the inclusion of internists should only occur as a last resort [14]. While this is certainly reasonable, we found we had occasional need for the services of internists because of prolonged duration of the disaster and therefore, response. Emergencies arising from the (internal) medical wards, in patients on admission prior to the crisis were also another instance that required the expertise of internists. Institutional response to a mass casualty situation is an effort that involves the entire hospital. Even non medically trained personnel could be utilized for simple interventions for patients with less severe injuries that would allow the experts to concentrate on those with critical injuries. Yasin et al. [15] found the mobilization of medical students as well as trained and untrained volunteers to be very useful in their response efforts to the mass casualty from the Pakistani earthquake of 2005 and that was our experience. These have to be properly supervised and guided otherwise it could introduce additional chaos that would be detrimental to the response effort [16].

## Conclusion

Frykberg points out that because of the rarity of true mass casualty incidents, experience from an actual event is the only reliable way to prepare for and implement the many unique elements of disaster response [17]. We have since incorporated most of the lessons learned from the Jos crisis of 2001 into our institutional preparedness for disaster response and indeed these have improved our response to three subsequent major crises in November 2008, January 2010 and December 2010. We point out that the plan should be tailored to the peculiarities of the environment and should anticipate the challenges posed by a crisis of prolonged duration. Fortunately, we have not had a crisis of similar duration or as destabilizing of organized societal mechanisms as this one since then, but we are guided by the dictum that *anything can happen anywhere, at any time.*

## Competing interests

Te authors declare that they have no competing interests.

## Authors’ contributions

KNO was involved in the mass casualty response, debriefings and drafted the manuscript. ICP was involved in the debriefings and conceptualization of the study. SJY was involved in the mass casualty response, debriefings, study design and literature search. AVR was involved in the debriefings and data collection. HCN was involved in the mass casualty response, debriefings and literature search. All authors read and approved the final manuscript.
